# Rbm24a Is Necessary for Hair Cell Development Through Regulating mRNA Stability in Zebrafish

**DOI:** 10.3389/fcell.2020.604026

**Published:** 2020-12-17

**Authors:** Yan Zhang, Yanfei Wang, Xuebo Yao, Changquan Wang, Fangyi Chen, Dong Liu, Ming Shao, Zhigang Xu

**Affiliations:** ^1^Shandong Provincial Key Laboratory of Animal Cells and Developmental Biology, School of Life Sciences, Shandong University, Qingdao, China; ^2^Department of Biomedical Engineering, Southern University of Science and Technology, Shenzhen, China; ^3^Key Laboratory of Neuroregeneration of Jiangsu and Ministry of Education, Co-Innovation Center of Neuroregeneration, School of Life Sciences, Nantong University, Nantong, China; ^4^Shandong Provincial Collaborative Innovation Center of Cell Biology, Shandong Normal University, Jinan, China

**Keywords:** Rbm24a, hair cells, inner ear, lateral line, mRNA stability

## Abstract

Hair cells in the inner ear and lateral lines are mechanosensitive receptor cells whose development and function are tightly regulated. Several transcription factors as well as splicing factors have been identified to play important roles in hair cell development, whereas the role of RNA stability in this process is poorly understood. In the present work, we report that RNA-binding motif protein 24a (Rbm24a) is indispensable for hair cell development in zebrafish. *Rbm24a* expression is detected in the inner ear as well as lateral line neuromasts. Albeit *rbm24a* deficient zebrafish do not survive beyond 9 days post fertilization (dpf) due to effects outside of the inner ear, *rbm24a* deficiency does not affect the early development of inner ear except for delayed otolith formation and semicircular canal fusion. However, hair cell development is severely affected and hair bundle is disorganized in *rbm24a* mutants. As a result, the auditory and vestibular function of *rbm24a* mutants are compromised. RNAseq analyses identified several Rbm24a-target mRNAs that are directly bound by Rbm24a and are dysregulated in *rbm24a* mutants. Among the identified Rbm24a-target genes, *lrrc23*, *dfna5b*, and *smpx* are particularly interesting as their dysregulation might contribute to the inner ear phenotypes in *rbm24a* mutants. In conclusion, our data suggest that Rbm24a affects hair cell development in zebrafish through regulating mRNA stability.

## Introduction

Hair cells are mechanosensitive sensory receptor cells in the inner ear and fish lateral line, characterized by the hairy-looking protrusions called hair bundles on their apical surface. The hair bundle of each hair cell consists of dozens to hundreds of actin-based stereocilia and one tubulin-based kinocilium. The stereocilia are organized into several rows of increasing height, with the mechano-electrical transduction (MET) channels localized at the tips of shorter row stereocilia (Beurg et al., 2009). The kinocilium is juxtaposed next to the tallest row stereocilia and plays an essential role in the development of hair bundle (Jones et al., 2008). As highly differentiated sensory receptor cells, hair cells are very sensitive to genetic mutations and environmental assaults, and deficits in hair cell development or function are the main reasons for hearing loss (Müller and Barr-Gillespie, 2015). Several key transcription factors such as Atoh1, Pou4f3, Gfi1, and Rfxs have been identified to play important roles in hair cell development (Erkman et al., 1996; Bermingham et al., 1999; Wallis et al., 2003; Elkon et al., 2015). At the post-transcriptional level, alternative splicing regulated by Srrm4, Sfswap, and Esrp1 has been shown to be involved in hair cell development (Nakano et al., 2012; Moayedi et al., 2014; Rohacek et al., 2017). However, the role of RNA stability regulation in hair cell development/function is less understood.

RNA-binding proteins (RBPs) play pivotal roles in post-transcriptional RNA processing, from pre-mRNA alternative splicing to mRNA stability, localization and translation (Hentze et al., 2018). Dysfunction of RBPs is associated with various types of developmental diseases (Brinegar and Cooper, 2016). RBPs usually bind to target RNAs through their so-called RNA-recognition motifs (RRMs). RNA-binding motif protein 24 (Rbm24) is an RBP that contains a single RRM and an alanine-rich low-complexity region (Fetka et al., 2000). It regulates alternative splicing through binding to the intronic splicing enhancer (ISE) sites in its target pre-mRNAs (Yang et al., 2014; Ito et al., 2016; Zhang et al., 2016; Lin et al., 2018; van den Hoogenhof et al., 2018; Liu et al., 2019). Moreover, Rbm24 regulates the stability or translation of its target mRNAs through binding to AU/U-rich elements (AREs; Jiang Y. et al., 2014; Xu et al., 2014; Zhang et al., 2018).

*In situ* hybridization results reveal that *Rbm24* is expressed in the otic vesicle during early embryonic development in zebrafish, frog, chick, and mouse (Fetka et al., 2000; Poon et al., 2012; Grifone et al., 2014; Maragh et al., 2014). Rbm24 is also expressed during later developmental stages of mouse inner ear (Cai et al., 2015; Grifone et al., 2018). Immunostaining and *in situ* hybridization reveal that Rbm24 is specifically expressed in the hair cells of embryonic and neonatal mice (Cai et al., 2015; Grifone et al., 2018). *Rbm24* expression in the developing mouse hair cells is further supported by the transcriptome data (Scheffer et al., 2015; Shen et al., 2015). The specific expression of Rbm24 in the otic vesicle during early development as well as in the hair cells at a later developmental stage suggests that Rbm24 might play important roles in the inner ear.

In the present work, we investigate the role of Rbm24 in hair cells using the zebrafish as a model. Zebrafish sensory hair cells are located in the five sensory epithelia (two maculae and three cristae) of the otic vesicle and the two lateral line systems (anterior lateral lines (aLL) and posterior lateral lines (pLL)) at the body surface (Nicolson, 2005). Each of the anterior and posterior macula is associated with a calcium carbonate-based otolith, and their hair cells sense sound and linear acceleration. Hair cells in the anterior, lateral and posterior cristae sense angular acceleration, whereas the lateral line hair cells sense water movement. Our present data suggest that inactivation of the *rbm24a* gene affects the development of hair cells in both the otic vesicle and the lateral line systems. Further investigations show that Rbm24a affects hair cell development through regulating the stability of its target mRNAs.

## Materials and Methods

### Zebrafish

All zebrafish animal procedures were carried out following the institutional guidelines approved by the Animal Ethics Committee of Shandong University School of Life Sciences. The *rbm24a* mutant zebrafish was generated using TALENs (transcription activator-like effector nucleases) as described previously (Shao et al., 2020). The *brn3c*:GFP transgenic zebrafish were generated as described previously (He et al., 2017).

### *In situ* Hybridization

Whole-mount *in situ* hybridization was carried out according to a standard protocol (Thisse and Thisse, 2008). For each target gene, a corresponding cDNA fragment was cloned into a pEASY Blunt Zero Cloning vector (Tiangen) and used as DNA template for synthesis of antisense RNA probe. The probes were labeled with digoxygenin-labeled rNTP mix (Roche Diagnostics), and NBT/BCIP was used as substrate. The probes for *dlx3*, *pax2a*, and *lrrc23* are the same as reported previously (Han et al., 2018; Xing et al., 2018). Primers for other probes are listed in Supplementary Table 1.

### Startle Response Measurement

Startle response was measured as described previously (Wang et al., 2017). Briefly, 10–20 zebrafish larvae at 5 dpf were maintained in an 8-cm Petri dish containing a thin layer (2 mm) of water. Tone bursts of 400 Hz at different sound intensity were delivered to the Petri dish through a mini vibrator (QY50R-Z). The movement of each larva was recorded using a digital camera (Basler acA1300–200 μm) at 120-frame per second (fps) and analyzed using a customized software developed in MATLAB (MathWorks, MA, United States). The distance of larvae’s C-shape movement upon sound stimulation was used as a measure of its auditory startle response.

### Vestibular Head Tilt Response Measurement

Vestibular head tilt response was measured as described previously (Sun et al., 2018). Briefly, individual zebrafish larva at 5 dpf was placed in a customized holder, where its tail was glued to immobilize the fish. The head of the fish was merged in the water for comfortable accommodation. The holder was then placed on a rotary platform with the fish head-up. The rotary platform was driven by a stepper motor (model TSM17Q-3AG, MOONS’, Shanghai, China) running in a sinusoidal profile of ±75 degrees around the vertical location. Larval eye movement stimulated by rotation was recorded using a monochrome IR camera (Point Gray, Richmond, Canada) at 30 fps and analyzed using a customized imaging analysis program written in MATLAB (Mathworks). The ratio of the maximum projection area change during eye movement to the maximum projection area of the eye in the larval frontal plane was used as a measure of the vestibular head tilt response.

### FM1-43FX Uptake Assay

Zebrafish larvae at 3 dpf were treated with 1.2 μM FM1-43FX (Molecular Probes, Invitrogen) in embryo medium for 30 s. After rinsing three times in fresh embryo medium, the larvae were anesthetized with 0.17 mg/mL^–1^ Tricaine (MS-222, Sigma) and the labeled lateral line hair cells were visualized using a florescent microscope (Olympus IX53).

### Paraffin Section and HE Staining

Embryos were anesthetized with 0.17 mg/mL^–1^ Tricaine (MS-222, Sigma), then fixed in 4% paraformaldehyde (PFA) at 4°C overnight. After that, embryos were embedded in paraffin and sliced into 5 μm-thick sections. Hematoxylin (Solarbio, H8070) and Eosin Y (Solarbio, G1100) staining was performed subsequently according to the manufacturer’s user guide. Images were taken using a light microscope (Leica DM2000).

### Confocal Microscopy

Embryos in *brn3c*:GFP transgenic background were anesthetized with 0.17 mg/mL^–1^ Tricaine (MS-222, Sigma), followed by fixation in 4% PFA at 4°C overnight. After washing with phosphate-buffered saline with Tween-20 (PBST), the samples were incubated with phalloidin (4 μg/ml, YEASEN, 40734ES80) at 37°C for 15 min. The samples were washed with PBST again, then mounted in 1% low-melting agarose. Images were taken using a confocal microscope (Zeiss, LSM700), and Z-stack projections were obtained by using the z-projection function. Image volumes were acquired at 0.5-μm intervals along the *z*-axis. Crista hair cells were imaged with a 0.95NA/20× Kort M27 objective lens. Neuromast hair cells were imaged with a 0.95NA/40× Kort M27 objective lens. Higher resolution images of crista and macula hair cells were taken with a 0.95NA/63× Kort M27 objective lens. The *x*-*y* pixel size is 1024 × 1024 for all images.

### Scanning Electronic Microscopy

Scanning electronic microscopy (SEM) was performed as previously described with modifications (Du et al., 2020). Briefly, embryos after 100% epiboly were treated with 0.06–0.08 g/L PTU (P110661, Aladdin Industrial Corporation, China) to block pigment synthesis. Then larvae at 72 hpf were anesthetized with 0.17 mg/mL^–1^ Tricaine (MS-222, Sigma) for 30 s and fixed with 2.5% glutaraldehyde at 4°C over night, followed by post-fixation with 1% osmium tetroxide at 4°C for 2 h. After dehydration in ethanol, samples were critically point dried using a Leica EM CPD300 (Leica, Germany), then mounted and sputter coated with 10-nm platinum using a Cressington 108 sputter coater (Cressington, United Kingdom). Images were taken using a Quanta250 field-emission scanning electron microscope (FEI, Netherlands) with a beam strength of 3 kV.

### RNAseq and RT-PCR

RNAseq analysis was performed by Genewiz (Suzhou, China) following the manufacturer’s protocols. Briefly, total RNAs were extracted from the otic vesicles of zebrafish larvae at 34 hpf (3 samples from wild-type and 3 samples from *rbm24a* mutants) using TRIzol reagent (Invitrogen), then mRNAs were enriched using the Poly(A) mRNA Magnetic Isolation Module (NEB). After mRNA fragmentation and cDNA synthesis, cDNA libraries were constructed using Ultra RNA Library Prep Kit for Illumina (NEB), followed by purification using Agencourt AMPure XP beads (Beckman) and quantification using Agilent 2100 (Agilent Technologies). The libraries were then multiplexed and clustered, and 150 bp paired-end sequencing was performed on Illumina HiSeq 2000. The sequencing results were filtered using Cutadapt and aligned with reference genome using Hisat2. Differential expression was analyzed using Htseq and DESeq2, and differential alternative splicing was analyzed using ASprofile and String Tie. RT-PCR and qPCR were then performed to confirm the sequencing results using primers specific for candidate genes (Supplementary Table 2).

### RNA Immunoprecipitation

RNA immunoprecipitation (RIP) analysis was performed as described previously (Li et al., 2020). Briefly, HEK293T cells were transfected with expression vectors using LipoMax transfection reagents (Sudgen, Cat. No. 32012) to express Rbm24a-Myc together with its target mRNAs. The transfected cells were lysed in lysis buffer containing 100 mM KCl, 5 mM MgCl_2_, 10 mM HEPES-NaOH, 0.5% NP-40, 1 mM DTT, 200 units/mL RNase inhibitor (Takara, Cat. No. 2313A), and EDTA-free protease inhibitor cocktail (Sigma-Aldrich, Cat. No. S8830). After centrifugation, the supernatant was collected and incubated with immobilized anti-Myc antibody (Sigma-Aldrich, Cat. No. E6654). The immunoprecipitated RNA was used as template for RT-PCR analysis using specific primers for Rbm24a-target mRNAs (Supplementary Table 3).

### Western Blot

The coding sequence (CDS) of *rbm24a* with or without the 8-bp deletion was inserted into modified pEGFP-N2 vector with EGFP-CDS replaced by Myc-CDS. HEK293T cells were transfected with expression vectors using LipoMax transfection reagents (Sudgen, Cat. No. 32012). 24 h after transfection, cells were lysed in ice-cold lysis buffer containing 150 mM NaCl, 50 mM Tris at pH 7.5, 1% (vol/vol) Triton X-100, 1 mM PMSF, and 1 × protease inhibitor cocktail (Roche). The supernatant was then collected after centrifugation and separated by polyacrylamide gel electrophoresis (PAGE), then transferred to PVDF membrane. After blocking in TBS (20 mM Tris–HCl, 150 mM NaCl) containing 5% non-fat dry milk and 0.05% Tween-20, the membrane was incubated with anti-Myc antibody (Abclonal, Cat. No. AE010) or anti β-Actin antibody (Abmart, Cat. No. P30002) at 4°C overnight, followed by incubation with HRP-conjugated secondary antibody (Bio-Rad, Cat. No. 170-6515 and 170-6516) at room temperature for an hour. The signals were detected with the ECL system (Thermo Fisher Scientific).

### Statistical Analysis

Each experiment was repeated at least three times. Student’s *t*-test was used to determine the statistical significance, and *p* < 0.05 was considered statistically significant. Data were shown as means ± SEM.

## Results

### *Rbm24a* Is Expressed in the Inner Ear and Lateral Line Neuromasts

**FIGURE 1:**
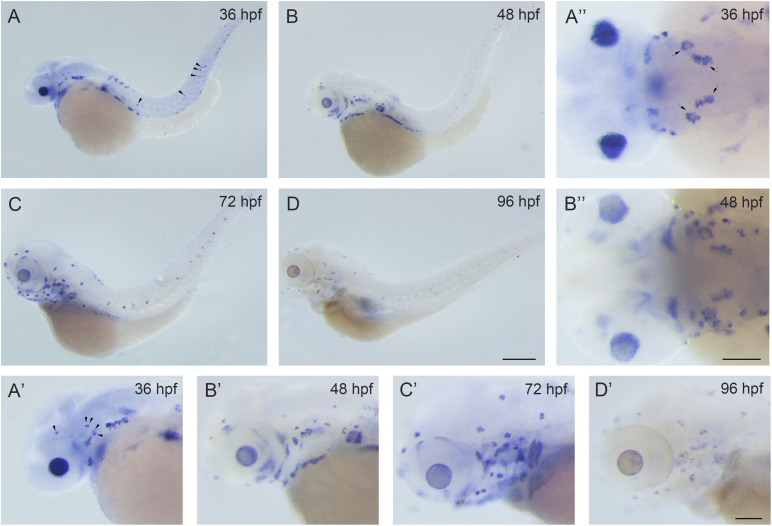
*Rbm24a* is expressed in the otic vesicles and lateral line neuromasts of the zebrafish. Expression pattern of *rbm24a* during development was examined by performing *in situ* hybridization of zebrafish embryos at 36 hpf **(A–A”)**, 48 hpf **(B–B”)**, 72 hpf **(C,C’)**, and 96 hpf **(D,D’)**. Otic vesicles are indicated with arrows, and lateral line neuromasts are indicated with arrowheads. Scale bars, 0.25 mm in **(A–D)**, 0.1 mm in **(A’–D’)** and **(A”,B”)**.

There are two *rbm24* homologs in the zebrafish, *rbm24a* and *rbm24b*. *Rbm24a* shows higher sequence homology with mammalian *Rbm24* compared to *rbm24b*. At the protein level, the similarity of mouse Rbm24 to zebrafish Rbm24a and Rbm24b is 87% and 72%, respectively. Meanwhile, *rbm24a* has been detected in the otic vesicles, whereas *rbm24b* has not (Poon et al., 2012; Maragh et al., 2014). Hence we focus on *rbm24a* in the present study. We first examined the spatial-temporal expression pattern of *rbm24a* by performing *in situ* hybridization. The results show that *rbm24a* transcripts could be readily detected in the otic vesicle and lateral line neuromasts at 36 hours post fertilization (hpf; Figures 1A,A’,A”). *Rbm24a* expression persists at 48 hpf and 72 hpf (Figures 1B–C’). By 96 hpf, *rbm24a* expression is still present in the otic vesicle and lateral lines (Figures 1D,D’). This specific expression pattern suggests that *rbm24a* might play an important role in the development of inner ear and lateral line neuromasts.

### *Rbm24a* Deficiency Affects Hair Cell Function

We investigated the function of *rbm24a* in hair cells using a *rbm24a* mutant zebrafish line, which contains a deletion of 8 base pairs (bp) in exon 1 of the *rbm24a* gene (Shao et al., 2020; Supplementary Figure 1A). The 8 bp-deletion will result in premature translational termination, giving rise to a truncated Rbm24a protein of only 28 amino acids (aa) instead of the full-length 230 aa (Supplementary Figure 1B). RT-PCR and *in situ* hybridization results show that *rbm24a* mRNA is still present in the homozygous *rbm24a* mutants (*rbm24a^–/–^*; Supplementary Figures 1C,D). There are several downstream AUG codons in *rbm24a* CDS, among which M59 is the first one (Supplementary Figure 1B). Translation started from these AUG codons in the mutant *rbm24a* mRNA might produce truncated Rbm24a proteins. Because Rbm24a-specific antibodies are not available, we expressed wild type or mutant Rbm24a with a Myc tag at the C-terminus in cultured HEK293T cells and examined their expression by performing western blot with anti-Myc antibody. From the cells expressing wild-type Rbm24a, we detected a band with a molecular weight of around 30 kDa. However, from the cells expressing mutant Rbm24a, we detected a band with a molecular weight of lower than 25 kDa, possibly corresponding to a truncated Rbm24a protein started from M59 (Supplementary Figure 1E). This mutant Rbm24a lacks most part of the RRM domain, suggesting that it is not functional. Consistent with this hypothesis, heterozygous *rbm24a* mutants are morphologically and behaviorally indistinguishable from wild type zebrafish, whereas homozygous *rbm24a* mutants suffer from severe heart defects and do not survive beyond 9 days post fertilization (dpf; Supplementary Figure 1F). Moreover, the body length of *rbm24a* mutants is slightly shorter than that of wild type (wild type at 48 hpf, 3.19 ± 0.02 mm; *rbm24a* mutant at 48 hpf, 2.85 ± 0.01 mm; wild type at 96 hpf, 3.55 ± 0.03 mm; *rbm24a* mutant at 96 hpf, 3.17 ± 0.02 mm. For each genotype, *n* = 18. *P* value < 0.000001).

**FIGURE 2:**
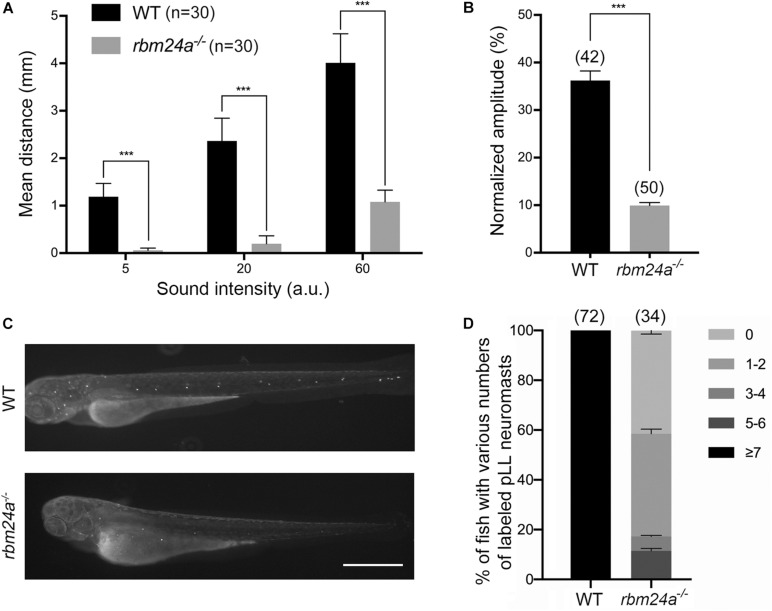
Hair cell function is affected in *rbm24a* mutants. **(A)** The auditory function of zebrafish larvae at 5 dpf is evaluated by examining the startle response to various sound intensity. ***p < 0.001. **(B)** The vestibular function of zebrafish larvae at 5 dpf is evaluated by vestibular head tilt response measurement. ***p < 0.001. **(C)** The function of hair cells is evaluated by performing FM1-43FX uptake experiment at 3 dpf. Scale bar, 0.5 mm. **(D)** The numbers of labeled pLL neuromasts per larvae are calculated according to the results from **(C)**. *P* value < 0.001. The numbers of larvae for each group are indicated in brackets.

We first evaluated the auditory function of mutant zebrafish larvae by measuring startle response (Kimmel et al., 1974; Zeddies and Fay, 2005). Various levels of sound stimulus were given to the fish larvae at 5 dpf, and the moving distance in a characteristic C-shape motion of the larvae was used as a measure of its auditory startle response. Compared to control wild-type larvae, *rbm24a* mutants show significant reduced startle response at each sound level, suggesting that *rbm24a* deficiency affects the auditory function of zebrafish (Figure 2A). We also evaluated the vestibular function of the mutants by measuring vestibular head tilt response (Mo et al., 2010). In this experiment, eye movement of 5 dpf larvae evoked by the rotational motion was used as a measure of the vestibular function. The results show that compared to wild-type control, *rbm24a* mutants show significantly reduced vestibular head tilt response, suggesting that *rbm24a* deficiency affects the vestibular function of zebrafish (Figure 2B).

Considering that *rbm24a* deficiency might affect muscle function, which could in turn affect the performance of the larvae in startle response and vestibular head tilt response, we then directly examined the functional integrity of hair cells by performing FM1-43FX uptake experiment. Florescent dye FM1-43 or its fixable analog FM1-43FX can enter hair cells through the MET channels, which provides an indicator of the functional integrity of hair cells (Gale et al., 2001; Meyers et al., 2003). The results show that FM1-43FX uptake by both aLL and pLL neuromasts is significantly decreased in 3 dpf *rbm24a* mutant larvae (Figures 2C,D). Taken together, our data suggest that hair cell function is severely affected in *rbm24a* mutant zebrafish.

### Early Inner Ear Development Is Largely Unaffected by *rbm24a* Deficiency

**FIGURE 3:**
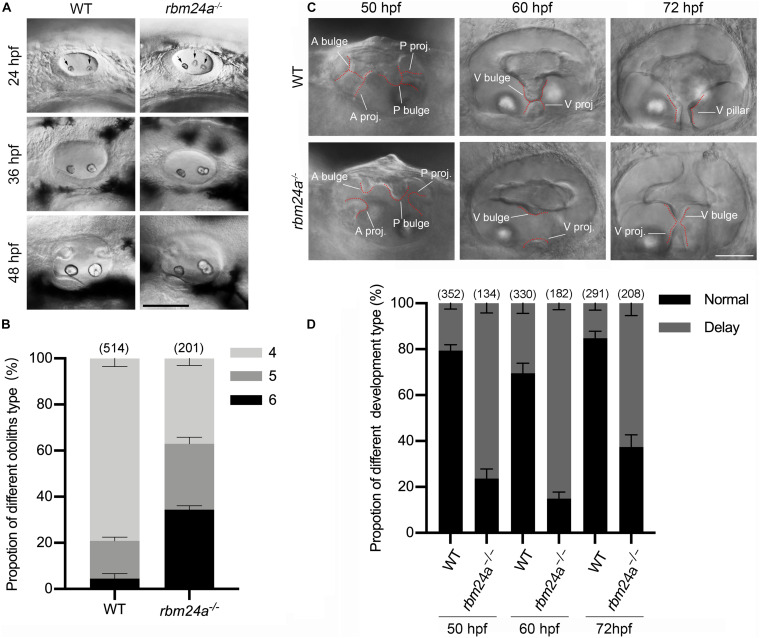
Otolith formation and semicircular canal fusion are delayed in *rbm24a* mutants. **(A)** Otolith formation in zebrafish larvae is examined using a light microscope at 24, 36, and 48 hpf as indicated. Otoliths are indicated with arrows. Scale bar, 0.1 mm. **(B)** The numbers of otic vesicles per larvae at 24 hpf are calculated according to the results from **(A)**. *P* values are 0.000833 (larvae with 4 otoliths), 0.020678 (larvae with 5 otoliths), and 0.00044 (larvae with 6 otoliths). **(C)** Fusion of the semicircular canals is examined using a light microscope at 50, 60, and 72 hpf as indicated. Scale bar, 50 μm. **(D)** The numbers of larvae with normal or delayed semicircular canal fusion are calculated according to the results from **(C)**. *P* values are 0.000001 (50 hpf), 0.00005 (60 hpf), and 0.000034 (72 hpf). The numbers of larvae for each genotype are indicated in brackets. A bulge, anterior bulge; A proj., anterior projection; P bulge, posterior bulge; P proj., posterior projection; V bulge, ventral bulge; V proj., ventral projection; and V pillar, ventral pillar.

Given the expression of *rbm24a* in the otic vesicles during early embryonic development, we wonder whether *rbm24a* deficiency affects early inner ear development. The morphology of otic vesicles was examined by performing hematoxylin-eosin (HE) staining, which does not reveal any significant difference between wild-type control and *rbm24a* mutants (Supplementary Figures 2A–C). However, examination with a light microscope shows that otolith formation in *rbm24a* mutants is delayed during early development. In each otic vesicle of early zebrafish embryo, otolith precursor particles are first distributed throughout the otic vesicle lumen, then tethered to form two otoliths at the otic vesicle poles, which are readily detectable by 24 hpf (Riley et al., 1997). Consistently, our data show that at 24 hpf, most wild-type embryos contain 4 otoliths (2 for each otic vesicle). In contrast, less than 40% of *rbm24a* mutants contain 4 otoliths, and all the others contain 5 or 6 otoliths (Figures 3A,B). The ectopic otolith gradually fuses to the regular posterior otolith, and there is no visible difference between the otoliths of the mutant and control larvae after 48 hpf (Figure 3A).

Moreover, delayed fusion of the semicircular canals is observed in *rbm24a* mutant larvae. The development of semicircular canals begins with the formation of three protrusions (the anterior, posterior, and lateral projections) from the otic vesicle between 42 and 48 hpf (Waterman and Bell, 1984). Afterward, the lateral projection bifurcates into the anterior and posterior bulges, which then fuse with the anterior and posterior projections to form the anterior and posterior pillars, respectively. Finally, the lateral projection gives rise to a ventral bulge, which then fuses with the newly developed ventral projection to form the ventral pillar by 72 hpf (Waterman and Bell, 1984). We found that in most wild-type larvae at 50 hpf, the anterior and posterior projections fuse with the anterior and posterior bulges, respectively, which is not observed in most *rbm24a* mutant larvae until 60 hpf (Figures 3C,D). Similarly, the ventral projection is fused with the ventral bulge in most wild-type larvae at 60 hpf, but not in most *rbm24a* mutant larvae until 72 hpf (Figures 3C,D).

**FIGURE 4:**
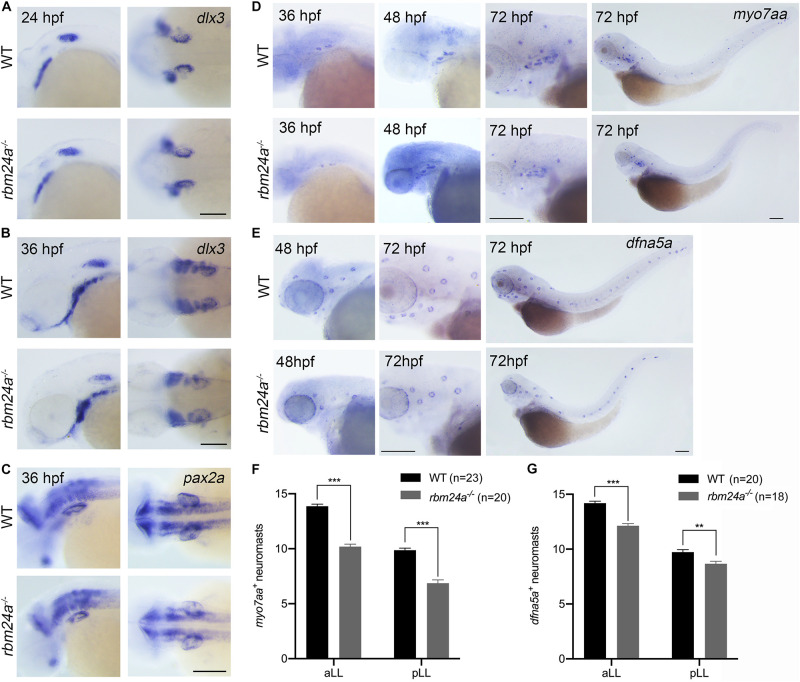
Early inner ear development is largely unaffected in *rbm24a* mutants. Inner ear development is examined by performing *in situ* hybridization of *dlx3* **(A,B)**, *pax2a* **(C)**, *myo7aa* **(D)**, and *dfna5a* **(E)** at different developmental stages as indicated. **(F)** The numbers of *myo7aa*^+^ neuromasts are calculated according to the results from **(D)**. **(G)** The numbers of *dfna5a*^+^ neuromasts are calculated according to the results from **(E)**. The numbers of larvae for each group are indicated in brackets. Scale bars, 0.2 mm. **p < 0.01; ***p < 0.001.

We then performed *in situ* hybridization to examine the expression of early otic vesicle markers in *rbm24a* mutants. *Dlx3* is expressed in the otic vesicle at an early developmental stage (Ekker et al., 1992). Our results show that *dlx3* expression in the otic vesicles of *rbm24a* mutant larvae is comparable to that of control at 24 and 36 hpf (Figures 4A,B). Similar results were obtained for another early otic vesicle marker, *pax2a* (Pfeffer et al., 1998; Figure 4C). Taken together, our present data suggest that early inner ear development is largely unaffected by *rbm24a* deficiency except for delayed otolith formation and semicircular canal fusion.

### *Rbm24a* Deficiency Leads to Reduced Hair Cell Number and Disorganized Hair Bundle

We moved on to examine the hair cells in *rbm24a* mutants by performing *in situ* hybridization of a hair cell marker *myo7aa* (Ernest et al., 2000). At 36 and 48 hpf, *myo7aa* is expressed in the otic vesicles of *rbm24a* mutant larvae similarly to that in controls (Figure 4D). By 72 hpf, robust expression of *myo7aa* in aLL and pLL neuromasts is detected in wild-type larvae; however, the number of *myo7aa*^+^ neuromasts is significantly decreased in *rbm24a* mutants (Figures 4D,F). The reduced expression of *myo7aa* in lateral lines of *rbm24a* mutants suggest that hair cell development is affected by *rbm24a* deficiency. We also performed *in situ* hybridization of *dfna5a*, a gene that is specifically expressed in the lateral line neuromasts (Jiang L. et al., 2014). The results show that the number of *dfna5a*^+^ neuromasts is slightly reduced in *rbm24a* mutants (Figures 4E,G), which could be explained by the shortened body length.

**FIGURE 5:**
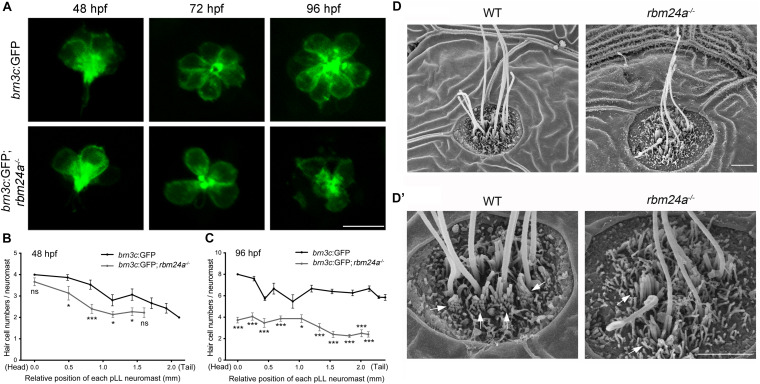
Development of pLL hair cells is affected in *rbm24a* mutants. **(A)** Confocal microscopic imaging analysis of pLL neuromasts in wild-type or *rbm24a* mutant *brn3c*:GFP line at different developmental stages as indicated. **(B,C)** The numbers of GFP-positive hair cells of each pLL neuromasts at 48 and 96 hpf are calculated according to the results from **(A)**. The numbers of larvae for each group is 15. **(D,D’)** Hair bundles of pLL neuromasts at 72 hpf are examined using SEM. Stereocilia are indicated with arrows. Scale bars, 10 μm in **(A)**, 2 μm in **(D,D’)**. ns, not significant; *p < 0.05; and ***p < 0.001.

To visualize the hair cells directly, we crossed *rbm24a* mutant zebrafish with *brn3c*:GFP transgenic zebrafish, which express GFP in the hair cells of both inner ear and lateral lines. The numbers of GFP-positive hair cells in each pLL neuromast are similar in *rbm24a* mutants and wild-type controls at 48 hpf (Figures 5A,B). However, by 72 hpf, the hair cell numbers per pLL neuromast in *rbm24a* mutants are less than that in controls, which becomes more obvious at 96 hpf (Figures 5A,C). At 96 hpf, there are 6–8 hair cells per pLL neuromast in wild-type controls, whereas no more than 4 hair cells per neuromast could be found in *rbm24a* mutants (Figures 5A,C). Meanwhile, similar to the *in situ* hybridization results of *dfna5a*, the total numbers of pLL neuromasts in *rbm24a* mutants are slightly less than that in controls (Figures 5B,C). Decreased hair cell numbers are also observed in the maculae and the cristae of the inner ear in *rbm24a* mutants (Supplementary Figures 3, 4).

**FIGURE 6:**
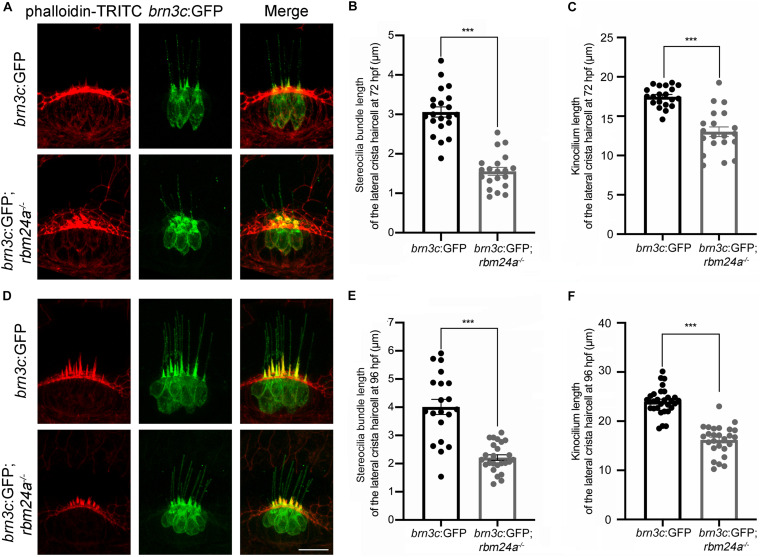
Development of lateral crista hair cells is affected in *rbm24a* mutants. Confocal microscopic imaging analysis of *rbm24a* mutant *brn3c*:GFP line at 72 hpf **(A)** and 96 hpf **(D)**. **(B,E)** Stereocilia length is calculated according to the results from **(A,D)**, respectively. **(C,F)** Kinocilia length is calculated according to the results from **(A,D)**, respectively. For stereocilia length measurement at 72 hpf, *brn3c*:GFP, *n* = 21 (from 3 larvae); *brn3c*:GFP;*rbm24a^– /–^*, *n* = 20 (from 4 larvae). For stereocilia length measurement at 96 hpf, *brn3c*:GFP, *n* = 20 (from 3 larvae); *brn3c*:GFP;*rbm24a^– /–^*, *n* = 25 (from 4 larvae). For kinocilia length measurement at 72 hpf, *brn3c*:GFP, *n* = 20 (from 3 larvae); *brn3c*:GFP;*rbm24a^– /–^*, *n* = 20 (from 4 larvae). For kinocilia length measurement at 96 hpf, *brn3c*:GFP, *n* = 30 (from 3 larvae); *brn3c*:GFP;*rbm24a^– /–^*, *n* = 26 (from 3 larvae). Scale bar, 10 μm. ***p < 0.001.

Scanning electrical microscopy was then employed to examine the morphology of hair bundles of the mutants. The results show that the stereocilia of pLL hair cells in wild type zebrafish are organized into a nice staircase pattern, which is largely lost in *rbm24a* mutants (Figures 5D,D’). To examine the hair bundles of inner ear hair cells, we used phalloidin to stain the F-actin core of stereocilia. Meanwhile, stereocilia and kinocilia could also be visualized by the strong GFP fluorescence in *brn3c*:GFP zebrafish. The results show that at 72 hpf, the length of hair cell stereocilia in *rbm24a* mutant crista is decreased to about half of that in wild-type control (Figures 6A,B). The length of kinocilia is also decreased in *rbm24a* mutants although to a lesser extent (Figure 6C). This phenotype remains in *rbm24a* mutants at 96 hpf (Figures 6D–F). Similar results are also observed in the macula (Supplementary Figures 5A–D). Taken together, our data suggest that *rbm24a* deficiency leads to reduced hair cell number and disorganized hair bundle.

### *Rbm24a* Deficiency Leads to Dysregulated Expression of Several Inner Ear-Expressed Genes

**FIGURE 7:**
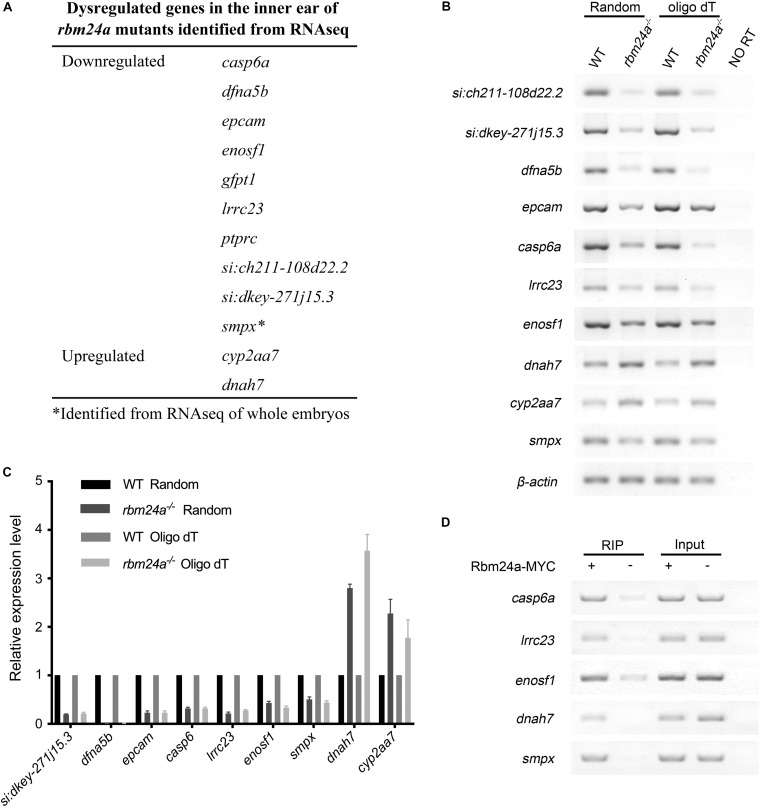
Dysregulated genes in the inner ear of *rbm24a* mutants. **(A)** Dysregulated genes in the inner ear of *rbm24a* mutants identified from RNAseq analysis. RT-PCR **(B)** and qPCR **(C)** were performed to confirm the RNAseq results. Random primers or oligo dT were used as primers for reverse transcription as indicated. **(D)** RIP experiments were performed to examine the binding of Rbm24a with its target mRNAs. Statistical analysis of the qPCR results shows that the expression difference is significant (*P* value < 0.000001, except that *P* value of *cyp2aa7* is 0.001315).

Previously, we performed RNA sequencing (RNAseq) of *rbm24a* mutant embryos to analyze the Rbm24a-regulated mRNAs (Shao et al., 2020). In the present work, we also prepared polyadenylated mRNA samples from the otic vesicle of *rbm24a* mutants at 34 hpf and performed RNAseq analysis. Neither of the analyses reveals meaningful differences in alternative splicing events between *rbm24a* mutants and controls. However, the RNAseq results reveal that the mRNA level of several genes such as *lrrc23*, *smpx*, *casp6a*, and *dfna5b* is significantly decreased in the *rbm24a* mutants (Figure 7A). On the other hand, the mRNA level of a few genes such as *cyp2aa7* and *dnah7* is upregulated in the *rbm24a* mutants (Figure 7A). The dysregulation was further validated by performing RT-PCR and qPCR (Figures 7B,C). Furthermore, RNA immunoprecipitation (RIP) experiments confirm that Rbm24a directly binds to these target mRNAs (Figure 7D).

**FIGURE 8:**
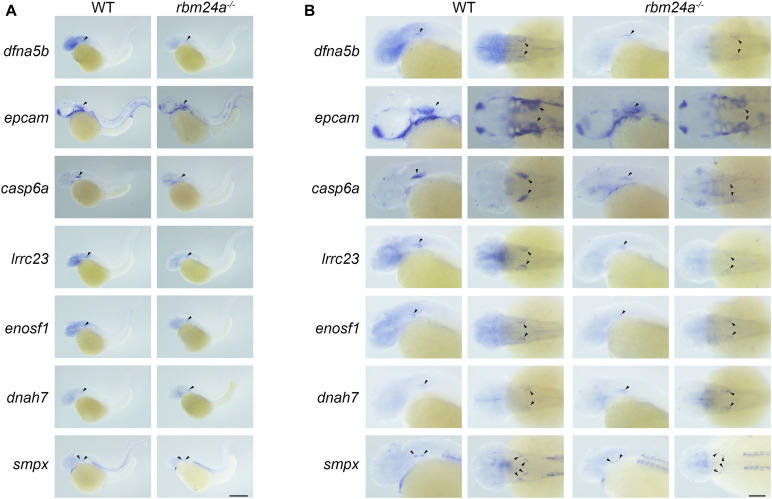
Expression pattern of important Rbm24a target mRNAs in the zebrafish. *In situ* hybridization of zebrafish embryos at 34 hpf was performed to examine the expression of *dfna5b*, *epcam*, *casp6a*, *lrrc23*, *enosf1*, *dnah7*, and *smpx*. **(A)** Lower magnification images. **(B)** Higher magnification images. Scale bars, 0.25 mm in **(A)** and 0.1 mm in **(B)**.

We then performed *in situ* hybridization to analyze the expression pattern of these Rbm24a-target mRNAs. The results show that the examined mRNAs are all expressed in the otic vesicles of wild-type controls (Figures 8A,B). Meanwhile, the expression of *dfna5b*, *epcam*, *casp6a*, *lrrc23*, *enosf1*, and *smpx* is decreased in the *rbm24a* mutants, whereas the expression of *dnah7* is increased in the mutants, which is consistent with the RNAseq, RT-PCR, and qPCR results (Figures 8A,B).

## Discussion

The physiological role of Rbm24 has been intensively investigated in the heart and skeletal muscles. *Rbm24* knockdown in C2C12 cells, *Xenopus*, or chick somatic myogenic progenitor cells affects myogenic differentiation (Miyamoto et al., 2009; Li et al., 2010; Grifone et al., 2014). *Rbm24* knockdown in zebrafish leads to deficits in cardiovascular development and somitogenesis (Maragh et al., 2011, 2014; Poon et al., 2012). Furthermore, inactivation of *Rbm24* in mice disrupts cardiac development through activating aberrant p53-dependent apoptosis (Yang et al., 2014; Zhang et al., 2018). Recently, we and others reported that Rbm24a plays pivotal roles in eye development of zebrafish through regulating mRNA stability and translation efficiency (Dash et al., 2020; Shao et al., 2020). In the present work, we report that Rbm24a is indispensable for hair cell development through regulating mRNA stability in zebrafish.

Expression of *rbm24a* could be detected in the otic visicles during early embryonic development. However, the morphology of otic visicle is largely unaffected in *rbm24a* mutants, suggesting that Rbm24a is dispensable for early inner ear development in the zebrafish. Nevertheless, otolith formation and semicircular canal fusion are delayed in *rbm24a* mutants. Otoliths are biomineralized aggregates of calcium carbonate and proteins sitting over hair cells in the maculae, embedded in the otolithic membrane (Schulz-Mirbach et al., 2019). Otolith formation in the zebrafish requires kinocilia and motile cilia, and defects in otolith formation is a characteristic phenotype of ciliary mutants (Stooke-Vaughan et al., 2012; Whitfield, 2020). For example, otolith formation is delayed in zebrafish deficient for *lrrc23* gene that encodes a radial spoke protein of the cilia (Han et al., 2018). Our data show that *lrrc23* mRNA is bound with Rbm24a and its level is decreased in *rbm24a* mutants, suggesting that Rbm24a might affect otolith formation through regulating *lrrc23* mRNA stability. Another potential Rbm24a-target that might contribute to the otolith phenotype is *dnah7*, which encodes dynein heavy chain 7, an inner arm component of cilia (Zhang et al., 2002). Interestingly, *dnah7* is one of the few genes whose expression is upregulated in *rbm24a* mutants.

Another important Rbm24a-target is *dfna5b*. Mutations in human *DFNA5* gene are associated with nonsyndromic hearing loss (Van Laer et al., 1998). There are two *DFNA5* orthologs in the zebrafish genome, *dfna5a* and *dfna5b*, among which *dfna5b* shows higher homology with human *DFNA5*. *Dfna5b* is expressed in the inner ear, and *dfna5b* knockdown leads to malformation of the semicircular canals (Busch-Nentwich et al., 2004). Given that both *rbm24a* mutation and *dfna5b* knockdown affect the development of semicircular canals, and that *dfna5b* expression is downregulated in *rbm24a* mutant larvae, we hypothesize that Rbm24a regulates semicircular canal development through affecting the stability of *dfna5b* mRNA. Meantime, *dfna5a* shows different expression pattern from *dfna5b*. Our *in situ* hybridization results show that *dfna5a* is expressed in a circular pattern at the lateral line neuromasts, and its expression level is largely unaffected in *rbm24a* mutants.

*Rbm24a* is also expressed in the hair cells of the inner ear and the lateral line neuromasts. Our data show that *rbm24a* deficiency results in decreased hair cell numbers and disorganized hair bundle, suggesting that Rbm24a is indispensable for hair cell development. Consistently, the MET function of hair cells is compromised in *rbm24a* mutants. As a result, both the auditory and vestibular function are affected in *rbm24a* mutants. RNAseq reveals that many inner ear-expressed genes are dysregulated in *rbm24a* mutants, which might contribute to the hair cell phenotypes. An interesting candidate is *smpx*, which is highly expressed in the hair cells of both mice and zebrafish (Yoon et al., 2011; Ghilardi et al., 2020). *SMPX* gene mutations are associated with nonsyndromic hearing loss DFNX4 (Huebner et al., 2011; Schraders et al., 2011). Smpx protein has been localized at the actin-based cuticular plate, a specialized structure at the apical surface of hair cells (Ghilardi et al., 2020). The cuticular plate is important for stabilizing the stereocilia, and its deficits could lead to stereocilia abnormalities (Pollock and McDermott, 2015; Du et al., 2019). Our present data suggest that Rbm24a might affect stereocilia development through regulating the stability of *smpx* mRNA. Further investigations are warranted to learn more about the underlying mechanism.

Our *in situ* hybridization results show that the number of *dfna5a*^+^ neuromasts is slightly decreased in *rbm24a* mutants. Similar pattern is also observed when GFP-positive neuromast hair cells are examined in *brn3c*:GFP background zebrafish. The slightly decreased pLL neuromast number could be explained by the shortened body length of *rbm24a* mutants. Rbm24a is indispensable for heart development, and *rbm24a* mutants suffer from severe heart defects and do not survive beyond 9 dpf (Shao et al., 2020). The severe overall sickness of *rbm24a* mutants might lead to the shortened body length before their eventual death. *In situ* hybridization results also reveal more significant decrease of *myo7aa*^+^ neuromast number in *rbm24a* mutants, which might be a result of reduced *myo7aa*^+^ hair cell numbers in each neuromast.

Rbm24 has been shown to be a major regulator of alternative splicing (Yang et al., 2014). Recently we showed that Rbm24 regulates the alternative splicing of *Cdh23* exon 68 in mice (Li et al., 2020). CDH23 forms the upper part of tip links that play a pivotal role in hair cell MET (Assad et al., 1991; Siemens et al., 2004; Kazmierczak et al., 2007). Mutations in *CDH23* gene have been associated with hearing loss in human, mice and zebrafish (Bolz et al., 2001; Bork et al., 2001; Di Palma et al., 2001; Sollner et al., 2004). However, our RNAseq analyses do not reveal any significant changes of alternative splicing in *rbm24a* mutants. We therefore performed RT-PCR experiments to examine the alternative splicing of *cdh23* gene in *rbm24a* mutants. The results show that the inclusion of *cdh23* exon 68 in *rbm24a* mutants is indeed decreased than that in wild type zebrafish (Supplementary Figures 6A,B). However, the remained *cdh23* exon 68 inclusion suggests that altered splicing of *cdh23* gene is not a main contributor to the auditory and vestibular phenotypes in *rbm24a* mutants.

In conclusion, our present work reveal that Rbm24a is an important post-transcriptional regulator in the hair cells, and that Rbm24a deficiency affects hair cell development in zebrafish through regulating RNA stability.

## Data Availability Statement

The original contributions presented in the study are included in the article/Supplementary Material, further inquiries can be directed to the corresponding author/s.

## Ethics Statement

The animal study was reviewed and approved by Animal Ethics Committee of Shandong University School of Life Sciences.

## Author Contributions

ZX: study concept and design. YZ, YW, XY, and CW: acquisition of data. YZ, FC, DL, MS, and ZX: analysis and interpretation of data. YZ and ZX: drafting the manuscript. MS and ZX: study supervision. All authors contributed to the article and approved the submitted version.

## Conflict of Interest

The authors declare that the research was conducted in the absence of any commercial or financial relationships that could be construed as a potential conflict of interest.
