# Supplementary material for: Rbm24a Is Necessary for Hair Cell Development Through Regulating mRNA Stability in Zebrafish
Source: Front Cell Dev Biol. 2020 Dec 17;8:604026. doi: 10.3389/fcell.2020.604026 (PMC7773828; doi:10.3389/fcell.2020.604026)
Supplement: Supplementary file 1 [file Data_Sheet_1.pdf]

# Rbm24a is necessary for hair cell development through regulating mRNA stability in zebrafish

Yan Zhang[1], Yanfei Wang[1], Xuebo Yao[1], Changquan Wang[2], Fangyi Chen[2], Dong Liu[3], Ming Shao[1,*], Zhigang Xu[1,4,*]

[1]Shandong Provincial Key Laboratory of Animal Cells and Developmental Biology, School of Life Sciences, Shandong University, Qingdao, Shandong 266237, China

[2]Department of Biomedical Engineering, Southern University of Science and Technology, Shenzhen, Guangdong 518055, China

[3]School of Life Science, Key Laboratory of Neuroregeneration of Jiangsu and Ministry of Education, Co-innovation Center of Neuroregeneration, Nantong University, Nantong, Jiangsu 226019, China

[4]Shandong Provincial Collaborative Innovation Center of Cell Biology, Shandong Normal University, Jinan, Shandong 250014, China

*Author for correspondence (shaoming@sdu.edu.cn; xuzg@sdu.edu.cn)

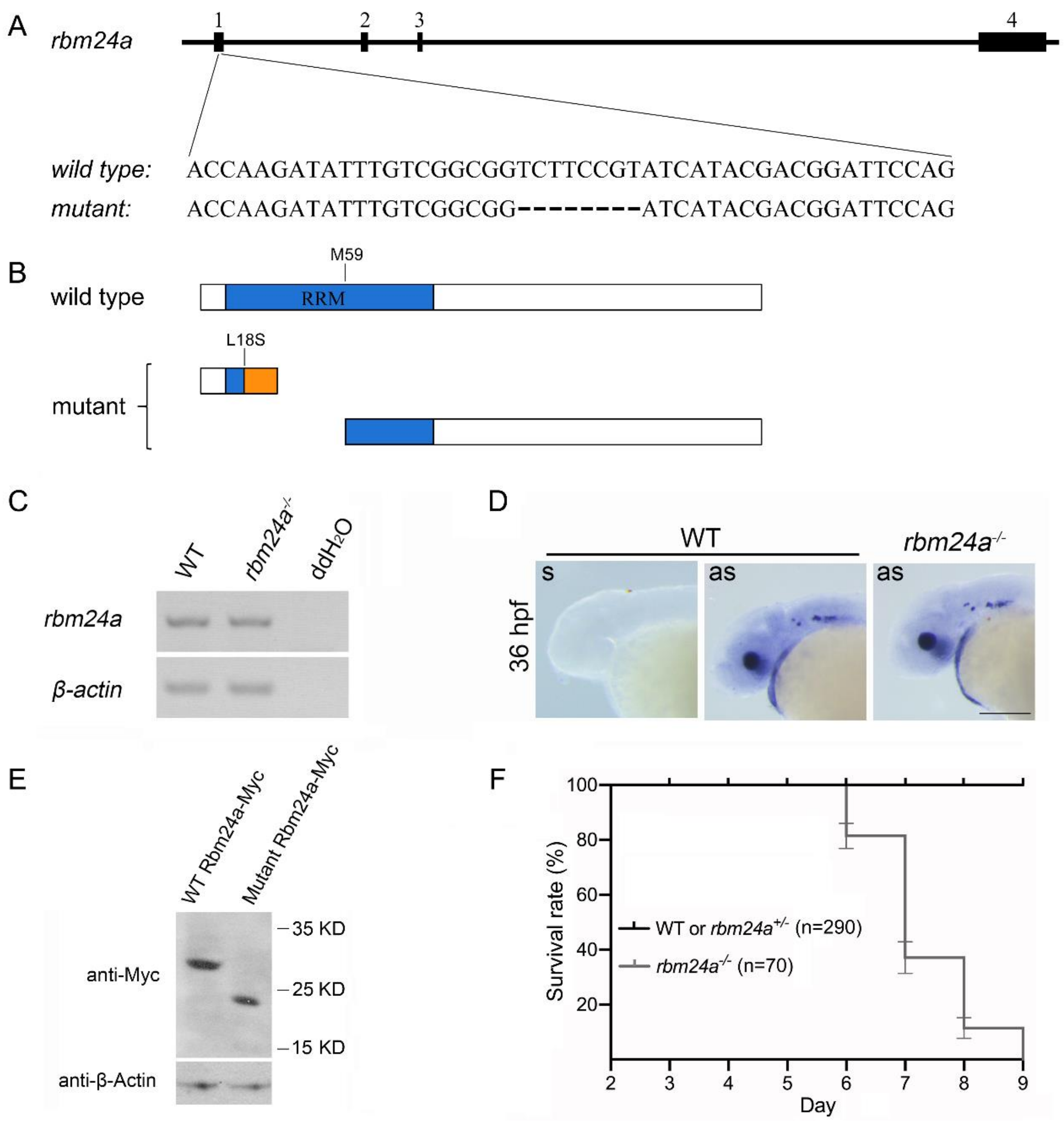


**Supplementary Figure 1.** Validation of *rbm24a* mutant zebrafish. (A) Schematic drawing of the genomic structure of zebrafish *rbm24a* gene. Exons are indicated with numbers. The *rbm24a* mutant zebrafish contains an 8-bp deletion in exon 1 of *rbm24a* gene. (B) Schematic drawing of the domain structure of zebrafish Rbm24a protein. Wild type Rbm24a protein contains 230 aa, whereas two potential mutant Rbm24a proteins contain 28 and 172 aa, respectively. (C) Total RNA was extracted from wild type or *rbm24a* mutant zebrafish larvae at 34 hpf and PCR was performed to examine the level of *rbm24a* mRNA. *β-actin* was used as an internal control. (D) *In situ*

hybridization was performed to examine the expression of *rbm24a* mRNA in wild type or *rbm24a* mutant zebrafish larvae at 36 hpf. s, sense probe; as, antisense probe. Scale bar, 0.2 mm. (E) The coding sequence (CDS) of *rbm24a* with or without the 8-bp deletion was inserted into expression vector to express wild type or mutant Rbm24a with a Myc tag at the C-terminus. Expression vectors were transfected into HEK293T cells and western blot was performed to examine their expression using an anti-Myc antibody. β-Actin was used as an internal control. (F) The survival rate of wild type or *rbm24a* mutant zebrafish. The numbers of larvae for each group are indicated in brackets.

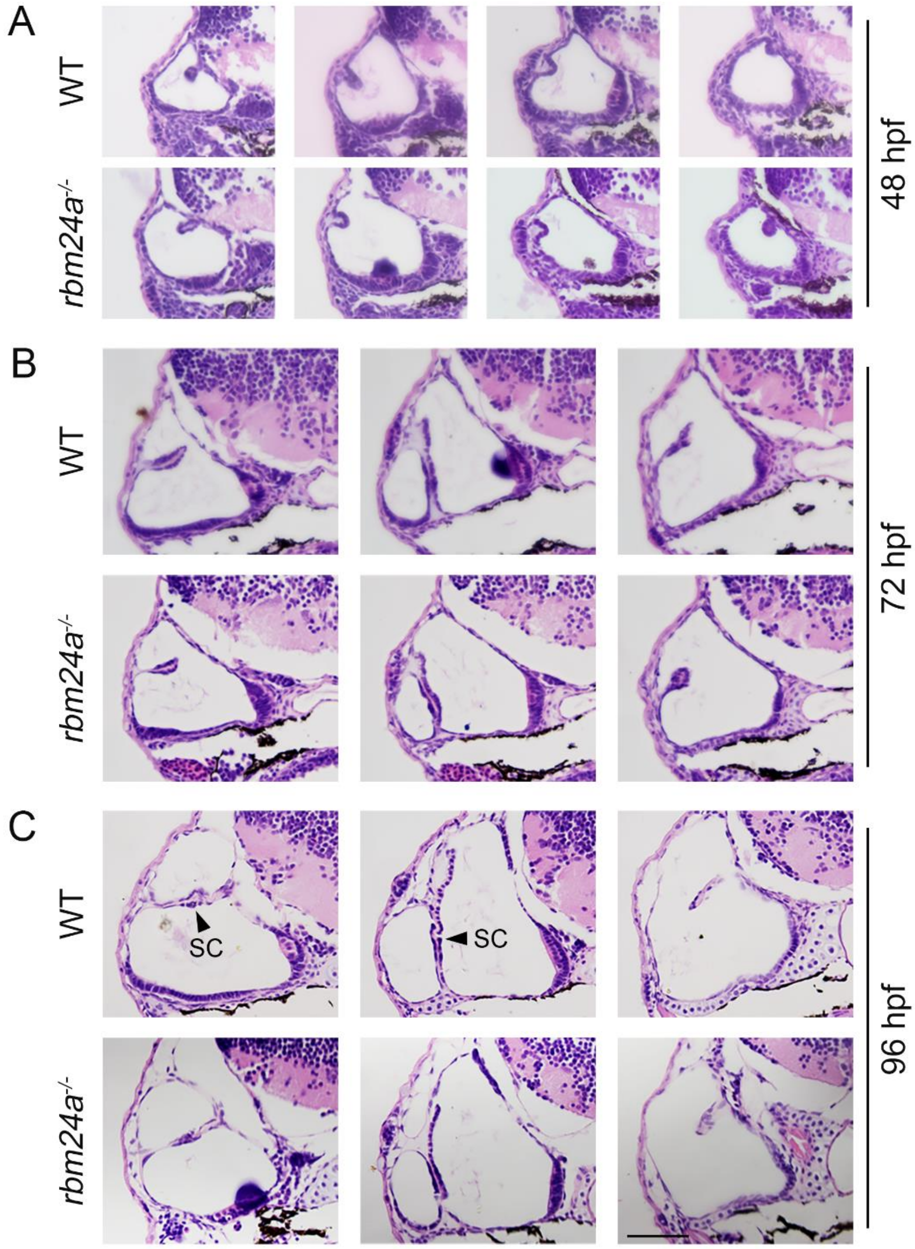


**Supplementary Figure 2.** Early inner ear development is largely unaffected by *rbm24a* deficiency. Cryosection and HE staining were performed to examine the inner ear morphology of wild-type or *rbm24a* mutants at 48 hpf (A), 72 hpf (B), and 96 hpf (C). SC, semicircular canal. Scale bar, 50 μm.

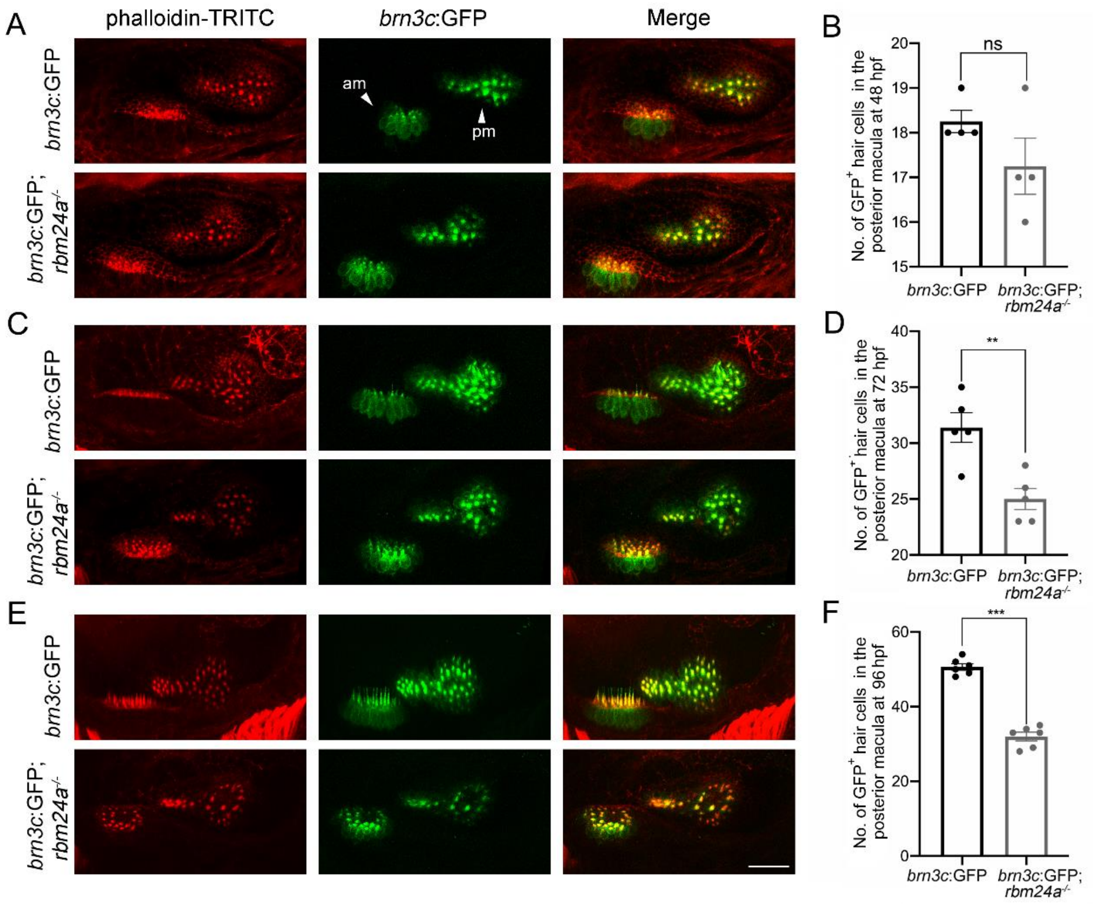


**Supplementary Figure 3.** Development of anterior and posterior macula hair cells is affected in *rbm24a* mutants. Confocal microscopic imaging analysis of *rbm24a* mutant *brn3c*:GFP line at 48 hpf (A), 72 hpf (C), and 96 hpf (E). The number of GFP-positive hair cells in the posterior macula at 48 hpf (B) (for each genotype, n=4), 72 hpf (D) (for each genotype, n=5), and 96 hpf (F) (for each genotype, n=6) is calculated accordingly. am, anterior macula; pm, posterior macula. Scale bar, 20 μm. ns, not significant; **, $p<0.01$; ***, $p<0.001$.

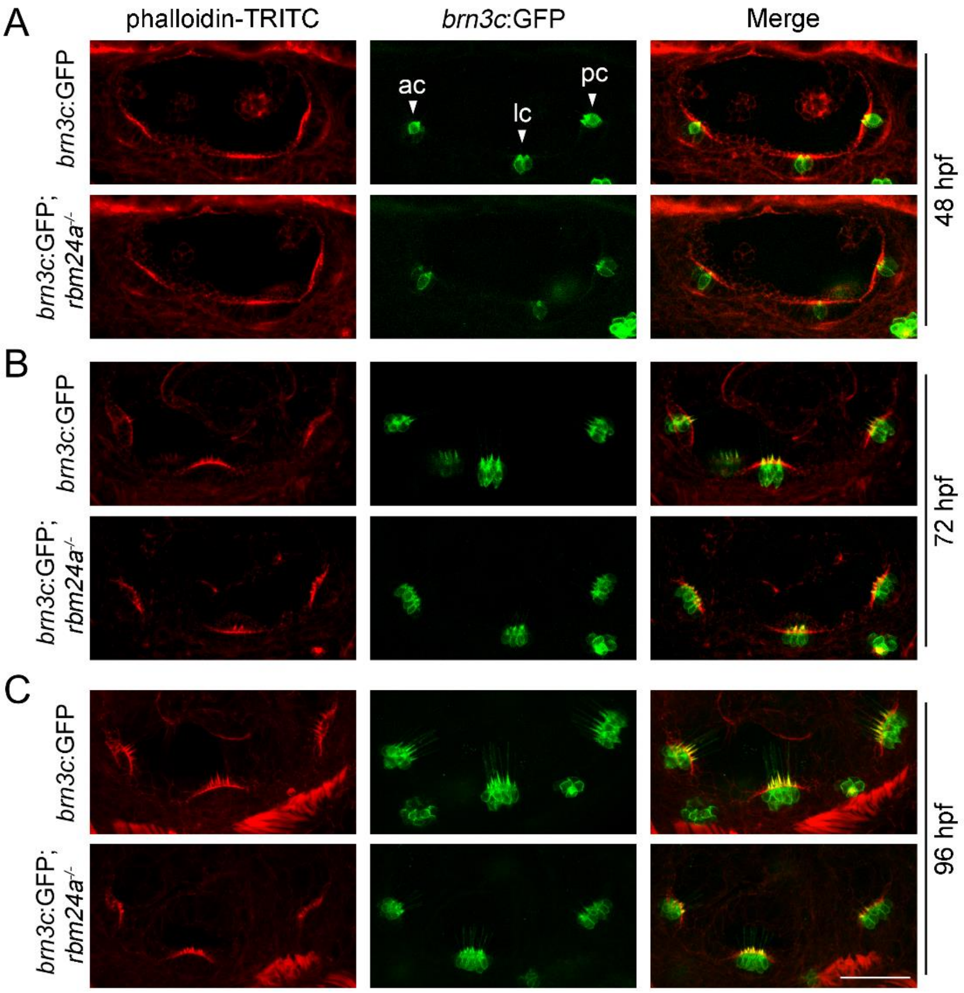


**Supplementary Figure 4.** Development of anterior, lateral, and posterior crista hair cells is affected in *rbm24a* mutants. Confocal microscopic imaging analysis of *rbm24a* mutant *brn3c*:GFP line at 48 hpf (A), 72 hpf (B), and 96 hpf (C). ac, anterior crista; lc, lateral crista; pc, posterior crista. Scale bar, 50 μm.

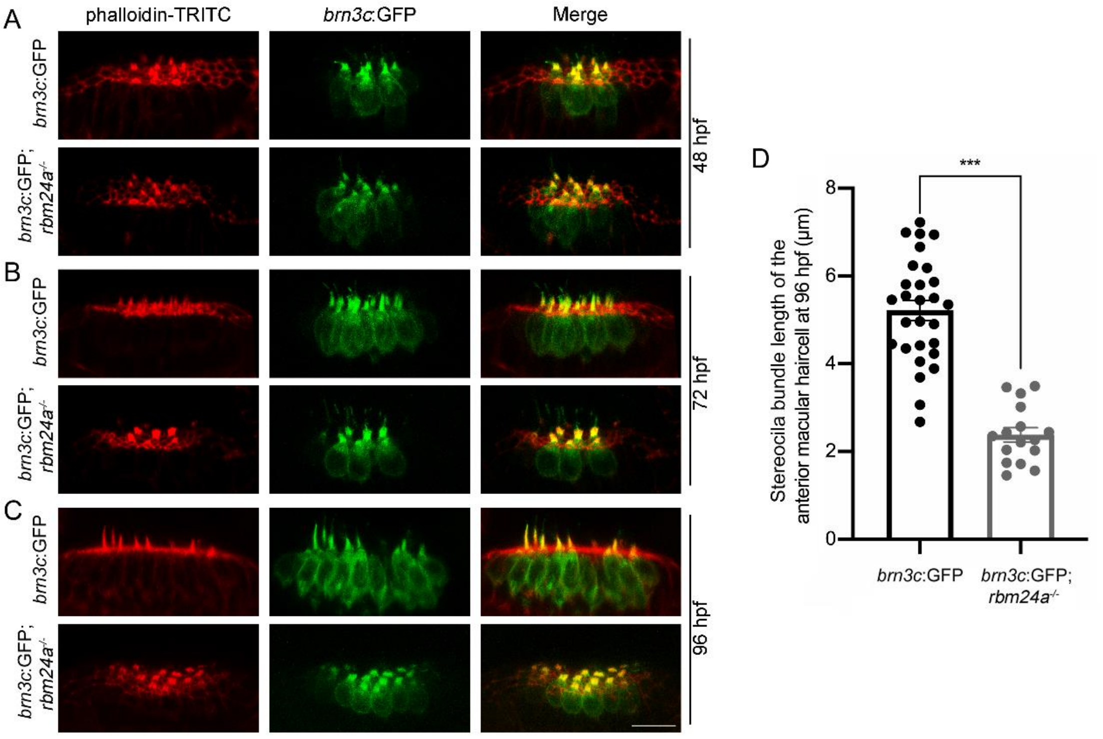


**Supplementary Figure 5.** Development of anterior macula hair cell is affected in *rbm24a* mutants. Confocal microscopic imaging analysis of *rbm24a* mutant *brn3c*:GFP line at 48 hpf (A), 72 hpf (B), and 96 hpf (C). (D) Stereocilia length of the anterior macula hair cells at 96 hpf is calculated according to the results from (C). For wild type larvae, n=28 (from 4 larvae); for *rbm24a* mutants, n=16 (from 3 larvae). Scale bar, 10 µm. ***, $p<0.001$.

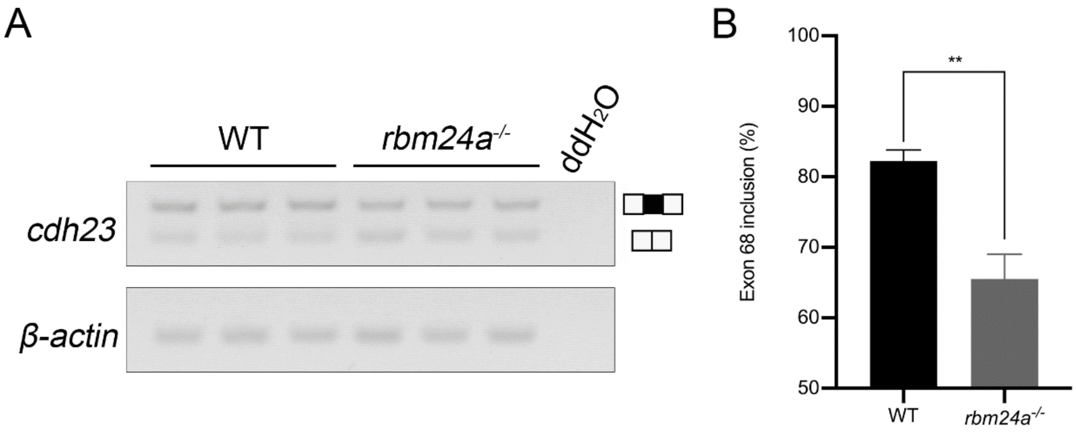


**Supplementary Figure 6.** Alternative splicing of *cdh23* exon 68 is affected in *rbm24a* mutants. (A) Total RNA was extracted from wild type or *rbm24a* mutant zebrafish larvae at 34 hpf and RT-PCR was performed to examine the alternative splicing of *cdh23* exon 68. *β-actin* was used as an internal control. (B) The inclusion rate of exon 68 in *cdh23* mRNA was calculated according to the results from (A). **, $p<0.01$.
